# A bibliometric review on vitamins and Alzheimer’s disease between 1996 and 2023

**DOI:** 10.3389/fnagi.2023.1144804

**Published:** 2023-05-11

**Authors:** Xiaoyu Sun, Haichun Xu, Huiling Qu, Wenwu Dong

**Affiliations:** ^1^Department of Neurology, The General Hospital of Northern Theater Command, Shenyang, China; ^2^Department of Psychiatry, Shenyang Jing’an Mental Health Hospital, Shenyang, China; ^3^Department of Thyroid Surgery, The First Hospital of China Medical University, Shenyang, China

**Keywords:** Alzheimer’s disease, bibliometric, CiteSpace, vitamins, Web of Science

## Abstract

**Objective:**

Alzheimer’s disease (AD) is a major disease that affects the elderly worldwide. Several studies have revealed that vitamins may influence the risk of developing AD. However, information in this field remains ambiguous. Therefore, this study aimed to examine the relationship between AD and vitamins, identify journal publications and collaborators, and analyze keywords and research trends using a bibliometric method.

**Methods:**

We systematically searched the Web of Science (WOS) Core Collection for papers published on AD and vitamins. Retrieved data regarding institutions, journals, countries, authors, journal distribution, keywords, and so on. SPSS 25 software was used for the statistical analysis, and CiteSpace V.6.1.R6 was used to visualize the information through collaborative networks.

**Results:**

A total of 2,838 publications were ultimately included in accordance with the specified inclusion criteria. The number of publications gradually increased from 1996 to 2023, with papers published in 87 countries/regions and 329 institutions. China (centrality: 0.02) and the University of Kentucky (centrality: 0.09) were the major research countries and institutions, respectively. NEUROLOGY was cited most frequently, reaching 1,573, and had the greatest impact. The cited keywords show that “Alzheimer’s disease,” “oxidative stress,” “vitamin E,” and “dementia” have been research hotspots in recent years. Beta-carotene emerged in 2023 and was identified as a developmental trend in this field.

**Conclusion:**

This is the first bibliometric analysis of vitamins associated with AD. We identified 2,838 articles in the field of vitamins and AD, analyzed the information of major countries/regions, institutions, and core journals in this field, and summarized the research hotspots and frontiers. These findings provide useful information for researchers to explore the role of vitamins in AD further.

## 1. Introduction

Alzheimer’s disease (AD) has significantly increased the global disease burden and is the leading cause of death among adults aged ≥ 65 years (Li et al., 2023; Nissim et al., 2023). In 2020, there were more than 50 million patients with AD worldwide (Zhang et al., 2021). AD is a progressive neurodegenerative disease that accounts for 80% of dementia cases (Ashraf et al., 2016; Graff-Radford et al., 2021; Scheltens et al., 2021). The emotional, physical, and financial toll of AD affects not only individuals and families, but also society broadly, and can only delay progress, but not cure it.

The mechanism of AD development has not been fully elucidated, although both genetic and environmental factors are involved (Cummings et al., 2022). The two hallmarks of AD are the extracellular deposits of neurotoxic β-amyloid (Aβ) in the brain and the accumulation of tau protein tangles in neurons, which lead to neuronal cell loss and damage to the vascular system, causing reduced blood flow to the brain and consequential cognitive impairment (Lei et al., 2021; Gabarro-Solanas and Urban, 2023; Wiatrak et al., 2023). Above all, oxidative stress has received considerable attention over the past several decades given its possible role in neurodegenerative processes (Chikara et al., 2018; Bai et al., 2022). It is a form of metabolic stress that emerges due to an imbalance between the production of reactive oxygen species (ROS) and the antioxidant mechanisms that counteract it (Rafnsson et al., 2013). Moreover, some studies have shown that oxidative stress plays an essential role in the development of AD by promoting Aβ deposition (Apelt et al., 2004; Simpson et al., 2010), tau hyperphosphorylation, and subsequent loss of synapses and neurons. To this end, active drug development strategies aim to reduce amyloid accumulation and toxicity (Aisen, 2005), slow tau phosphorylation and tangle formation (Fillit and Refolo, 2002), and promote neuronal survival and synaptic function (Yan et al., 2013; Tonnies and Trushina, 2017).

Currently, four drugs being used to treat AD. Three of them are acetylcholinesterase inhibitors, which inhibit the breakdown of acetylcholine after nerve stimulation, and NMDA antagonists, which block glutamate receptors (Cummings et al., 2019; Breijyeh and Karaman, 2020). Additionally, vitamins are involved in neurogenesis, neuronal defense mechanisms, metabolic responses, neuronal survival, and neuronal transmission. According to the current knowledge, vitamins may influence the risk of developing AD. Their deficiency can lead to functional brain abnormalities such as oxidative stress, mitochondrial dysfunction, protein accumulation (synaptic nucleoproteins and A-beta plaques), neurodegeneration, and excitatory toxicity (Kumar et al., 2022; Andrews et al., 2023). Research has shown that a diet rich in antioxidant vitamins can slow the AD progression. A meta-analysis showed that the intake of vitamins A, B, C, D, and E may decrease the risk of AD (Jayedi et al., 2019).

Different vitamins have different mechanisms of action in AD. In animal experiments, vitamin C deficiency impaired cognition, increased amyloid accumulation and deposition, and increased oxidative stress (Dixit et al., 2015). Nevertheless, vitamin C supplementation reduces spatial learning deficiency in APP/PSEN1mice and amyloid-beta oligomerization without affecting plaque formation (Harrison et al., 2009), and increased levels of Hcy promote DNA damage, which sensitizes neurons to Aβ toxicity (Kruman et al., 2002). The role of dietary folate in providing the methyl groups required for the maintenance and modulation of DNA methylation makes it a nutrient of interest in AD. Several lines of evidence regarding the beneficial properties of vitamin B12 with respect to AD pathogenesis can also be found in cell culture and *in vitro* studies (Lauer et al., 2022). The positive effects of vitamin B12 on AD pathology are associated with amyloid formation and fibrillization, epigenetic modifications, tau fibrillization, synaptogenesis of neuronal membranes, oxidative stress, and cholesterol synthesis (Moore et al., 2012). In recent years, VK2 has become an essential nutrient for human health, and an increasing number of studies suggest that VK2 may play an important role in slowing or even preventing the progression of AD by reducing Aβ-induced apoptosis and limiting oxidative stress to improve neuronal health (Popescu and German, 2021). Vitamin D (VD) also exerts protective effects against ROS and reactive nitrogen species, preventing oxidative damage and reducing the cytotoxicity induced by amyloid plaques (Jayedi et al., 2019; Jia et al., 2019). Vitamin E plays an important role in protecting cells against harmful free radicals and enhancing the immune response in elderly people, especially the α-tocopherol form. There is sufficient evidence from laboratory and animal studies to consider it of potential interest as a treatment for patients with AD (Ibrahim et al., 2017; Kryscio et al., 2017; Lloret et al., 2019). Therefore, when preventing AD, it is recommended that a diet rich in vitamins A, D, C, K2, B12, and folic acid and higher doses of vitamin E be supplemented. However, some studies suggest that the amount of fruits and vegetables will supplement the content of antioxidant vitamins, which are usually ineffective in patients with dementia. Further research should focus on solving these ambiguous problems (Mielech et al., 2020).

As a new discipline since its formal establishment, bibliometric methods have tracked the progress and contributions of clinicians and basic scientists in improving diagnosis and treatment. In this study, we summarized recent, highly cited studies, countries with the highest contributions, and published research profiles on the relationship between vitamins and AD over the past decade using bibliometric methods. Bibliometric analysis is a tool used for the statistical examination of scientific publications. They mainly help identify past and future trends in certain research topics (Kokol et al., 2021). They also allow researchers to conduct a more in-depth analysis of the cooperation between authors and countries and to understand the impact of scientific publications in the research community (Kutluk and Danis, 2021). Although some bibliometric analyses of AD have been published (Serrano-Pozo et al., 2017; Dong et al., 2019; Trejo-Castro et al., 2022), none of these studies examined the relationship between AD and vitamins. As it is one of the most novel and powerful theories in the development of AD pathology, it is important to discover future trends and guide researchers in carrying out new research.

Previous reviews only relied on individual research through literature summary and extraction, so they could not fully reflect the time and space distribution of researchers, institutions, and journals. In addition, it is difficult to visualize the internal structure of the knowledge base and the research focus, and few systematic, comprehensive, or visual studies have been conducted. Therefore, this study aimed to comprehensively analyze the current situation, research focus, and development trends of AD and vitamin research through a bibliometric analysis of 2,838 publications on AD and vitamins published from 1996 to 2023. These findings may help follow-up researchers study the relationship between AD and vitamins, identify journal publications and collaborators, and analyze keywords and research trends, thus promoting research aimed at determining the cause, mechanism, and treatment of the disease.

## 2. Materials and methods

### 2.1. Search strategy

**FIGURE 1:**
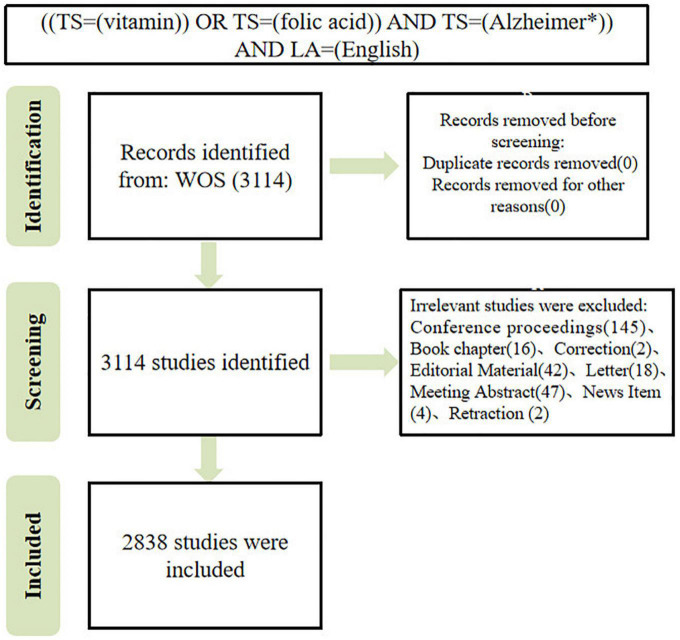
Flow diagram of the publications screening process.

The data used in this study were collected from the Science Citation Index Expanded (SCI-E) of the Web of Science (WOS) Core Collection database. The search terms used to identify publications included the following topics: (“Alzheimer disease” or “Alzheimer’s disease”) and topics: (“vitamin” or “folic acid”) and (“English”). The search strategy identified publications with words mentioned in their titles, abstracts, author keywords, or keywords. Only articles and reviews were included in this review. Timespan: 1 January 1996 to 17 March 2023 (publication date), and the date of retrieval was 17 March 2023. All publications from the search were preliminarily included, and we screened all publications by reading the title, abstract, and author keywords and excluded irrelevant literature (papers not officially published, book chapters, editorial material, conference abstracts and proceedings and corrigendum documents, unrelated papers). Discrepancies were observed through discussions. A PRISMA 2020 flow diagram (Page Matthew et al., 2021) depicting the flow of information through the different phases of the analysis can be found in Figure 1.

### 2.2. Analysis methods and tools

The selected documents retrieved by the WOS were imported into the plain text format “txt” files containing all records and references, named “download_XXX” and saved. CiteSpace V.6.1.R6 was used to perform bibliometric analysis, calculate centrality, and draw co-occurrence maps of countries/regions, institutions, authors, published journals, cited literature, keyword co-occurrence maps, cluster maps, time graphs, and emergent maps. Parameter setting: the overall selected time span was from January 1996 to March 2023. The slice length was set to 1 year. Select “Pruning,” “Pathfinder” and “Pruning network,” and save the default settings for other options. In the visualization diagram, node and font size represent the frequency, the lines between nodes (edges) represent the relationship between nodes, the thickness of the line represents the closeness of the relationship, and the colors of nodes and lines represent the distance between years.

## 3. Results

### 3.1. Publication trends

**FIGURE 2:**
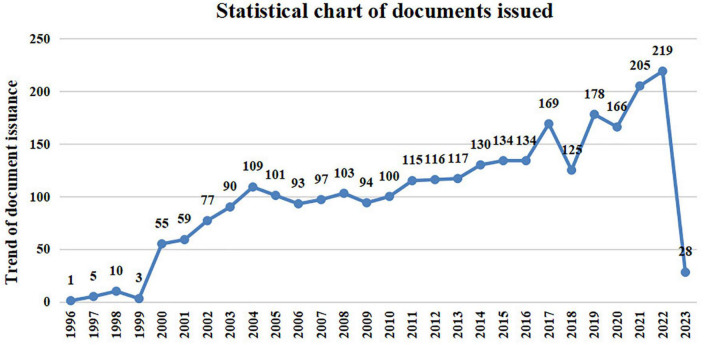
Annual number and growth trend of publications from 1996 to 2023.

A total of 2,838 publications met the inclusion criteria. Researchers are paying increasing attention to AD each year. Consistent with this, despite minor fluctuations, there was an overall upward trend in the number of studies on vitamin A intervention in AD. As shown in Figure 2, publications in this field began in 1996, but the number of publications was relatively small, and the annual number of publications in this field has steadily increased since 2000, reaching a peak in 2017 (169 publications), 2019 (178 publications), and 2022 (219 publications) and is expected to continue to increase in 2023. The large number of publications and literature citations in recent years has attracted the attention of scholars in this area.

### 3.2. Bibliometric analysis of countries/regions, institutions, and author

Literature was obtained from 87 countries/regions. As shown in Table 1, among all the countries/regions where the literature was published, the top three in terms of the number of publications were: China (922), the United States (873), and Italy (215). Centrality is an indicator of the importance of the nodes in a network. Centrality > 0.1 indicates more cooperation between nodes and greater contribution. In the centrality ranking, the United States (0.24) ranked first, followed by England (0.22) and Australia (0.20), indicating that the main research countries in this field are the United States and England, and that these countries have close cooperation. The centrality of China is 0.02, indicating that it has a high number of publications but lacks in-depth cooperation with the international community, which should be further strengthened in the future.

**TABLE 1:** Top 10 countries/regions in frequency/centrality of publications.

Rank	Frequency	Country	Centrality	Country
1	922	China	0.24	United States
2	873	United States	0.22	England
3	215	Italy	0.20	Australia
4	184	England	0.13	Germany
5	179	Germany	0.12	Tunisia
6	126	France	0.10	Japan
7	113	Australia	0.09	India
8	111	Canada	0.08	Russia
9	109	Japan	0.08	Ukraine
10	106	India	0.07	Italy

**FIGURE 3:**
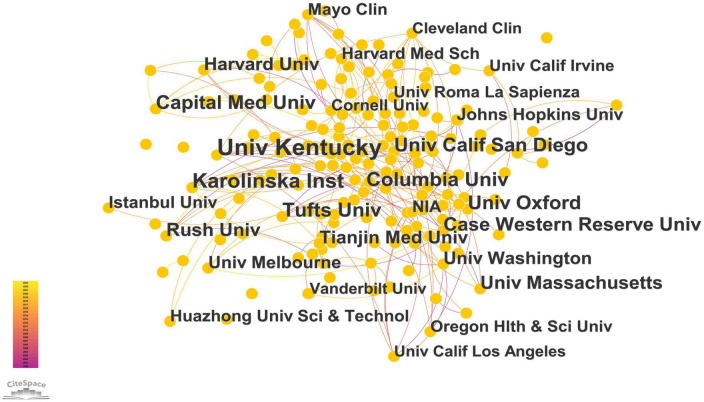
The map of co-institutions. The nodes represent institutes, the number of lines represents the intensity of cooperation between institutions.

Table 2 and Figure 3 list the 329 institutions that published literature in this field. The most productive institution is the University of Kentucky in the United States (50 publications), followed by the Karolinska Institute in Sweden (30 publications) and Tufts University in the United States (28 publications). Columbia University in the United States (24 publications) was ranked fourth. The University of Cambridge topped the list for centrality (0.12), followed by the University of California San Diego (0.10), the University of Kentucky (0.09), and Rush University (0.09). The Capital Medical University of China ranked eighth (0.07).

**TABLE 2:** Top 10 institutions in frequency/centrality of publications.

Rank	Frequency	Institution	Centrality	Institution
1	50	Univ Kentucky	0.12	Univ Cambridge
2	30	Karolinska Inst	0.10	Univ Calif San Diego
3	28	Tufts Univ	0.09	Univ Kentucky
4	24	Columbia Univ	0.09	Rush Univ
5	23	Univ Calif San Diego	0.08	Case Western Reserve Univ
6	23	Univ Oxford	0.08	NIA
7	22	Capital Med Univ	0.08	UCL
8	21	Univ Massachusetts	0.07	Capital Med Univ
9	20	Case Western Reserve Univ	0.07	King Abdulaziz Univ
10	20	Rush Univ	0.06	Univ Oxford

**FIGURE 4:**
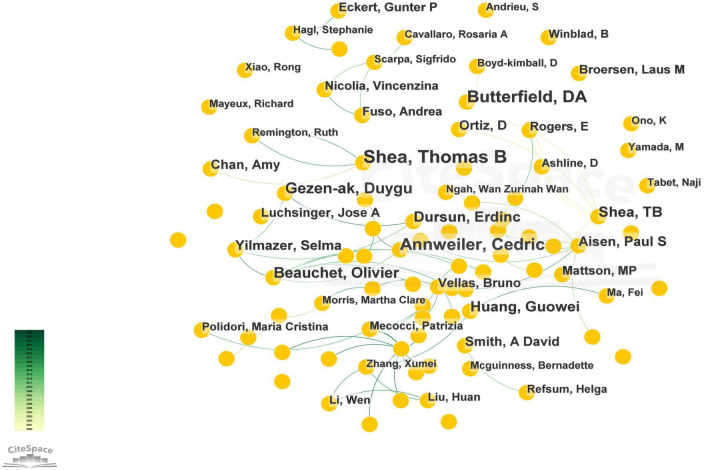
The map of co-authors. The nodes in the map represent co-authors, and lines between the nodes represent co-citation relationships.

Overall, 576 authors contributed to the study. From the nodes and lines of Figure 4, it can be seen that the core authors of this research field are Shea and Thomas B, who published 23 papers, ranking first in this field, followed by Annweiler and Cedric, who published 19 papers, and Butterfield and DA, who published 18 papers. In addition, it can be seen that a large number of author cooperation teams led by Shea and Thomas B, as well as other authors’ research cooperation teams in this field, are presented in the form of connections between nodes, and most of the three or more cooperative teams. There have been a few independent studies. The nodes with more connections represent the author’s greater contribution and influence in the academic network.

### 3.3. Bibliometric analysis of journals and journals’ co-citations

A total of 39 journals participated in the publication of the relationship between vitamins and AD. As shown in Table 3, the largest published amount was NEUROLOGY, which was cited the most frequently, reaching 1,573 times, and had the greatest impact. J ALZHEIMERS DIS was the second, having been cited 1,308 times. The remaining journals with more than 1,000 citations are listed in the following order: P NATL ACAD SCI USA was cited 1,265 times, NEUROBIOL AGING was cited 1,248 times, ARCH NEUROL-CHICAGO was cited 1,234 times, AM J CLIN NUTR was cited 1,208 times, J BIOL CHEM was cited 1,138 times, and J NEUROSCI and NEW ENGL J MED were cited 1,133 times, J NEUROCHEM was cited 1,028 times.

**TABLE 3:** Top 10 journals in frequency/centrality of publications.

Journal	Citations	Countries/ regions	Centrality
NEUROLOGY	1,573	United States	0.37
J ALZHEIMERS DIS	1,308	Netherlands	0.62
P NATL ACAD SCI USA	1,265	United States	0.22
NEUROBIOL AGING	1,248	England	0.17
ARCH NEUROL-CHICAGO	1,234	United States	0.08
AM J CLIN NUTR	1,208	United States	0.12
J BIOL CHEM	1,138	United States	0.04
J NEUROSCI	1,133	United States	0.08
NEW ENGL J MED	1,133	United States	0.1
J NEUROCHEM	1,028	England	0.37

The top ten analysis of citation counts in this field are shown in Table 4. The first list with 581 citations is “Plasma homocysteine as a risk factor for dementia and Alzheimer’s disease” by Seshadri S, published in NEW ENGL J MED in 2002. The second is “Vitamin E and donepezil for the treatment of mild cognitive impairment” published by the Petersen RC team in NEW ENGL J MED in 2005, cited 123 times. The third is the Littlejohns TJ team, whose 2014 publication in NEUROLOGY, “Vitamin D and the risk of dementia and Alzheimer disease,” cited up to 119 times. Fourth, Dysken MW’s team, their 2014 publication, “Effect of vitamin E and memantine on functional decline in Alzheimer disease: the TEAM-AD VA cooperative randomized trial” published in JAMA, was cited 105 times. The fifth was the Engelhart MJ team, which was published in JAMA in 2002.

**TABLE 4:** Top ten cited references.

Citations	References	First author	Journal	Year
174	Plasma homocysteine as a risk factor for dementia and Alzheimer’s disease	Seshadri S	NEW ENGL J MED	2002
123	Vitamin E and donepezil for the treatment of mild cognitive impairment	Petersen RC	NEW ENGL J MED	2005
119	Vitamin D and the risk of dementia and Alzheimer disease	Littlejohns TJ	NEUROLOGY	2014
105	Effect of vitamin E and memantine on functional decline in Alzheimer disease: the TEAM-AD VA cooperative randomized trial	Dysken MW	JAMA	2014
95	Dietary intake of antioxidants and risk of Alzheimer disease	Engelhart MJ	JAMA	2002
90	Dietary intake of antioxidant nutrients and the risk of incident Alzheimer disease in a biracial community study	Morris MC	JAMA	2002
85	High-dose B vitamin supplementation and cognitive decline in Alzheimer disease: a randomized controlled trial	Aisen PS	JAMA	2008
75	Folate, vitamin B12, and serum total homocysteine levels in confirmed Alzheimer disease	Clarke R	ARCH NEUROL-CHICAGO	1998
75	Effect of 3-year folic acid supplementation on cognitive function in older adults in the FACIT trial: a randomized, double blind, controlled trial	Durga J	LANCET	2007
73	Preventing Alzheimer’s disease-related gray matter atrophy by B-vitamin treatment	Douaud G	P NATL ACAD SCI USA	2013

### 3.4. Bibliometric analysis of keywords

The difference in node size represents the difference in keyword frequency. The larger the node, the higher in the keyword frequency. As shown in Table 5, the top five keywords for frequency were Alzheimer’s disease (1,423 times), oxidative stress (678 times), vitamin E (536 times), dementia (391 times), and mild cognitive impairment (317 times). The top five keywords for centrality were Alzheimer’s disease (0.57), vitamin E (0.31), oxidative stress (0.24), dementia (0.16), and plasma homocysteine (0.14).

**TABLE 5:** Top ten keywords in cited times or centrality.

Rank	Citations	Keyword	Centrality	Keyword
1	1,423	Alzheimer’s disease	0.57	Alzheimer’s disease
2	678	Oxidative stress	0.31	Vitamin E
3	536	Vitamin E	0.24	Oxidative stress
4	391	Dementia	0.16	Dementia
5	317	Mild cognitive impairment	0.14	Plasma homocysteine
6	280	Alzheimer disease	0.13	Brain
7	256	Vitamin D	0.13	Vitamin c
8	254	Risk	0.13	Free radical
9	236	Brain	0.11	Risk
10	215	Folic acid	0.11	Risk factor

**FIGURE 5:**
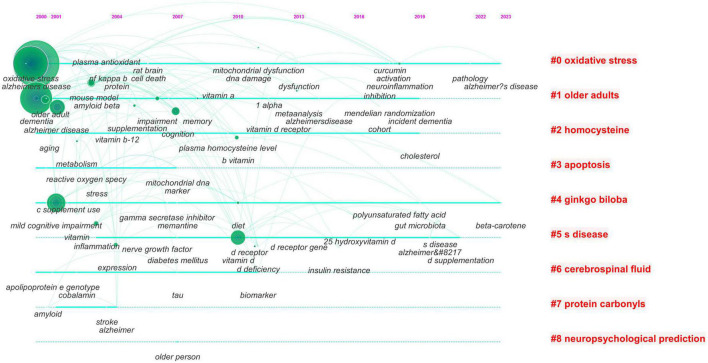
The time diagram of keywords.

Based on keyword co-occurrence, CiteSpace can perform cluster analysis on keywords using a logarithmic likelihood ratio algorithm. Keyword clustering is a network of related keywords to similar research topics in the research field, and each cluster is identified by the title appearing frequently in the publication. Clusters are numbered from #0; the smaller the number, the more keywords they contain. The lines between the nodes within the cluster represent the co-occurrence relationships. The more lines, the higher the co-occurrence degree between keywords in this field In this study, the clustering module value (modularity, Q value) was 0.4043 > 0.3 means the cluster structure was significant, and the average clustering contour value (Silhouette, S value) was 0.7687 > 0.7, indicating that clustering was more reliable. As shown in Table 6, the top nine categories of cluster labels were oxidative stress, older adults, homocysteine, apoptosis, ginkgo biloba, s disease, cerebrospinal fluid, protein carbonyls, and neuropsychological prediction. According to Figure 5, these clusters are closely connected and intertwined, indicating a close relationship between them. Therefore, cross-cluster research in these fields could be a new entry point for research.

**TABLE 6:** Keyword cluster.

Cluster ID	Cluster label	Size	Centrality	Year	Label (LLR)
#0	Oxidative stress	60	0.795	2005	Oxidative stress; cytotoxicity; dementia; b27 supplement; cytosolic free calcium concentration
#1	Older adults	54	0.659	2005	Older adults; dementia; tocopherol; cognition; aged
#2	Homocysteine	45	0.789	2002	Homocysteine; cardiovascular disease; folate; cobalamin; b vitamin
#3	Apoptosis	42	0.757	2001	Apoptosis; beta-amyloid; neuronal cells; calcium influx; neuroprotection
#4	Ginkgo biloba	20	0.758	2004	Ginkgo biloba; supplements; donepezil; cholinesterase inhibitor; double blind
#5	S disease	19	0.836	2012	S disease; vitamin d; Alzheimer; oxidative stress; schizophrenia
#6	Cerebrospinal fluid	16	0.815	2001	Cerebrospinal fluid; lipoproteins; oxidation; nutritional status; elderly population
#7	Protein carbonyls	6	0.917	2002	Protein carbonyls; malonaldehyde dimethyl acetal; morphology; fructo-oligosaccharides; a beta oligomers
#8	Neuropsychological prediction	2	0.992	2007	Neuropsychological prediction; trial; older person; e epsilon 4 allele; questionable dementia

The time graph takes the historical process as the carrier, marks the important keywords in the past on the axis, the node size represents the frequency, and visualizes the changes in the important keywords with time. There were also nineclusters, as shown in Figure 5. These keywords were spread out in the cluster according to the year in which they appeared, showing the development of keywords in each cluster. In 2010, many hot keywords appeared in related fields, and there were more connections during the outbreak period of research, and the heat of research tended to be stable. By 2023, words such as beta-carotene had emerged and become the focus of the field.

### 3.5. Future research direction analysis

**FIGURE 6:**
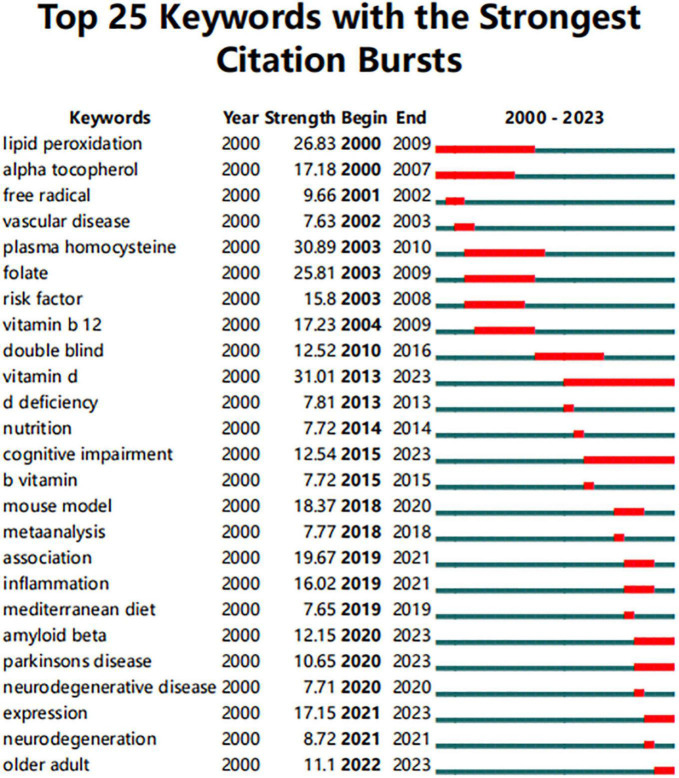
The emergent map of keywords. The red horizontal stripes represent the years with the most frequent keywords. The green horizontal stripes represent the years with the most infrequent keywords.

Emergent words refer to keywords that burst out suddenly in a certain period of time, which can detect the decline or rise of a keyword. As Figure 6 shows, there were no emergent keywords between 1996 and 2000. However, by 2000, the “lipid peroxidation” had started to appear and decreased until 2009. In the same year, the hot word “alpha tocopherol” appears and the heat persists until 2007. The emergence times for these two keywords were longer. In addition, vitamin d (emerging in 2013), cognitive impairment (emerging in 2015), amyloid beta (emerging in 2020), Parkinson’s disease (emerging in 2020), expression (emerging in 2021), and older adults (emerging in 2022) will all last until 2023, indicating that these words are still hot research topics in the field.

## 4. Discussion

The purpose of this study was to conduct a bibliometric analysis of publications on vitamins and AD over the past two decades. To the best of our knowledge, this is the first study to summarize the developmental status, research hotspots, and future trends of vitamin intake and AD. Our research is of great significance to students, researchers, and clinicians working in this field. In the past two decades, vitamin research has attracted increasing attention, and more importantly, its relationship with disease. However, AD is the subject of a long-term study that lacks success in improving diagnostic accuracy or the possibility of treatment. According to our findings, the pathological mechanism by which vitamins affect AD may be related to the antioxidant activity pathway and protein expression affecting the apoptotic pathway. A large number of scientific publications have recently been published regarding vitamins and AD. In our search, from 2,838 scientific contributions in the field, since 1996, the number of publications has increased annually, and by 2022, the annual number of publications will reach 219. According to the data provided, it is almost certain that this trend will continue until 2023.

In all studies, 10 highly cited publications were published in the past two decades, which may be a period of high-quality development in the field of vitamin and AD research. By analyzing journals related to vitamins and AD, we determined that researchers are concentrated in neuroscience and geriatrics. Among them, NEUROLOGY (1,573 citations), J ALZHEIMERS DIS (1,308 citations), P NATL ACAD SCI USA (1,265 citations), NEUROBIOL AGING (1,248 citations), and ARCH NEUROL-CHICAGO (1,234 citations) have paid the most attention to this field, demonstrating that the research field has focused on neuroscience and gerontology. Seven of the top ten journals come from the United States, indicating its leading role in this field.

Unsurprisingly, most of the research originated in the United States. We found that China and the United States were the leaders in terms of the number of publications, with 922 and 873 publications, respectively. Italy ranked third, with 215 publications and 184 publications in England. The distribution of institutions is scattered. Some institutions in China, such as Capital Medical University and Tianjin Medical University, have participated in research in this field and have made several achievements. Capital Medical University topped the list with 22 publications, and Tianjin Medical University with 17 publications. The University of Kentucky in the United States (50 publications), the Karolinska Institute in Sweden (30 publications), and Tufts University in the United States (28 publications) are productive institutions in this field. The analysis in terms of centrality indicated that the University of Cambridge was the most influential research institution.

Author analysis revealed a network of core author collaborations in the field of vitamin D and AD research. Since 1996, 576 researchers have participated in publications in this field. Shea, Thomas B from the Department of Biological Sciences in United States was undoubtedly the most influential and contributing author in this field. However, the publication “Plasma homocysteine as a risk factor for dementia and Alzheimer’s disease” is the most cited publication in this field, which verified that plasma homocysteine level are a strong, independent risk factor for the development of dementia and Alzheimer’s disease (Seshadri et al., 2002). However, research directions are relatively scattered. The top seven authors in terms of centrality were all smaller than 0.1, indicating that these publications need more in-depth study by researchers in this field.

According to the analysis of the co-citation count among the 10 references, most were human experiments, and four were randomized controlled trial (RCTs). The number of citations can be regarded as a relatively reasonable indicator to evaluate the quality of publications, which indicates that vitamins D, E, and B are the focus of current research, but recent research shows that vitamin C supplementation is a feasible strategy for prevention and treatment of AD (Hamid et al., 2022), Vitamin C treatment restored Aβ induced abnormal metabolites, and the role of antioxidants in the repair of AD metabolic disorder provide a new metabonomic observation and explanation (Zhang et al., 2022). Thus, VK2 may also play an important role in the prevention and treatment of AD (Popescu and German, 2021). Increasing evidence shows that there is a relationship between VK2 and AD, but no clinical study has investigated this relationship in humans. To fill this gap in clinical research, it is important to study any possible relationship between VK2 levels and AD risk through observational studies and RCTs (Popescu and German, 2021). Vitamin A may inhibit Aβ oligomerization (Ono and Yamada, 2012). These studies showed that although vitamins D, E, and B are traditionally considered to prevent AD, vitamins A, K, and C should also attract more attention in the future for their application potential.

The top 10 co-occurrence times of keywords are listed in Table 5 showing that “Alzheimer’s disease,” “oxidative stress,” “vitamin E,” “dementia”, “mild cognitive impairment,” and “vitamin D” directions are very significant in accordance with the citation counts. Both of them focused on vitamins D and E. This result showed that researchers have paid more attention to vitamins D and E than to other vitamins. These results also show that vitamins and AD require more innovation than other vitamins such as vitamins A, B, and C. Centrality represents the influence of a keyword in the network. The highest centrality is 0.57 for the keyword “Alzheimer’s disease,” which suggests that these studies need more attention in the future. Figure 5 presents a cluster diagram of keywords. The clusters included oxidative stress, older adults, homocysteine, apoptosis, ginkgo biloba, s disease, cerebrospinal fluid, protein carbonyls, and neuropsychological prediction. Cluster 0 contained five keywords that focused on the role of animal experiments in AD; the frequently mentioned keywords were cytotoxicity, dementia, b27 supplement, and cytosolic free calcium concentration. This cluster indicates that experimental research on AD is the focus of current research in this field. Recently, neuropsychological prediction (Cluster #8) has received increased attention, indicating the transformation process of experimental research achievements to clinical applications (Bastin and Salmon, 2014).

Although the trend of publishing this topic will certainly increase in the next few years, bibliometric analysis enables us to explore the trend theme, and we found that gut microbiota and inflammation are the research frontiers since 2019, indicating that gut microbiota has aroused great interest since 2019 (Jiang et al., 2017; Ozben and Ozben, 2019). Bibliometric articles published in 2022 showed that the number of publications on the gut microbiome and AD will increase exponentially by 2020. Nearly 400 related publications will be published in 2021, and the number will be close to 700 by 2022 (Trejo-Castro et al., 2022). In addition to providing vitamins through the diet, the gut microbiome is a source of vitamins for the host. Studies on animals and humans have shown that microorganisms can synthesize vitamins C, K, and B vitamins (Steinert et al., 2020). Another research trend is inflammation, which is considered an event in the progression of AD as the main therapeutic target of AD. The pathogenesis of AD, including cytokines and chemokines, the complement system, oxidative stress, and the cyclooxygenase pathway, is related to neuroinflammation in the AD brain (Rather et al., 2021). The vitamin B complex (folic acid, vitamin B6, and vitamin B12) and vitamin D3 can enhance Aβ clearance to prevent AD progression and exert anti-inflammatory effects (Szczechowiak et al., 2019). Low B12 levels are associated with increased IL-6 production by peripheral blood mononuclear cells (Politis et al., 2010). Vitamin A, Vitamin C, and Vitamin E also have a role as an anti-inflammatory agent (Reifen, 2002; Morris, 2004).

## 5. Limitations

Some scientific considerations should be taken into consideration when interpreting the results of this study. First, these publications only come from the WOS Core Collection database and do not record references to textbooks or other databases. Second, our analysis only included English publications, which may have resulted in an incomplete search.

## 6. Conclusion

To the best of our knowledge, this is the first time that a bibliometric analysis has been conducted on vitamins for AD. We identified the 2,838 most cited publications in the field of vitamins and AD and analyzed the information of major countries/regions, institutions, and core journals in this field, and summarized research hotspots and frontiers. We believe that these findings will provide useful information for researchers to further explore the role of vitamins in AD.

## Data availability statement

The original contributions presented in this study are included in the article/supplementary material, further inquiries can be directed to the corresponding authors.

## Author contributions

HQ and WD contributed to the conception and design of the study and revised the manuscript. HX collected and analyzed the data. XS wrote the manuscript. All authors read and approved the final version of manuscript.
